# Perception of reduced forms in English by non-native users of English

**DOI:** 10.3389/fpsyg.2024.1305134

**Published:** 2024-04-24

**Authors:** Malgorzata Kul

**Affiliations:** Department of Contemporary English and Multilingualism, Adam Mickiewicz University, Poznań, Poland

**Keywords:** reduced forms, speech perception, E-prime, English as a second language (ESL), corpus-based study, Polish learners

## Abstract

The article reports the results of a study on the perception of reduced forms by non-native users of English. It tests three hypotheses: (i) reduced forms with context are recognized more accurately and faster than reduced forms without context; (ii) gradient reduction is perceived less robustly than the categorical one; and (iii) subjects with musical background perceive reduced forms better than those without. An E-Prime study on 102 Polish learners of English was implemented, comparing participants’ accuracy and reaction times with a control group of 14 native speakers. The study was corpus-based and used 287 reduced forms from a corpus of Lancashire. The results indicate that (i) lexical context and phone density significantly affect perception, (ii) the category of reduction process (gradient or categorical) is irrelevant, and (iii) musical background only partially impacts non-native perception.

## 1 Introduction

Due to a low degree of formality, attention (Labov, 1994), specific audience design (Bell, 1984, 2001), and high speech rate, casual speech abounds in reduction processes affecting both vowels and consonants. This, in turn, results in reduced forms (Shockey, 2003; Johnson, 2004). For instance, the phrase *I do not know /aɪ doʊnt noʊ/* assumes the reduced form */ dənoʊ/.*

Reduced forms, the topic of the study, are phonetic and phonological deviations from citation forms: “rapid speech—different word forms can emerge from rapid speech when compared with slow speech. For example, *perhaps* in clearly articulated slow speech becomes *praps* in rapid speech” (Bussmann, 1996, p. 396). According to Shockey (2003), “there are some phonological differences from citation forms […] I call these differences reductions” (Shockey, 2003, p. 1–2). Hanique et al. (2013) defined reduced forms as follows: “in conversational speech […] segments may be very short, altered […] or even completely absent” (Hanique et al., 2013, p. 1,644). One may understand reduced forms as a result of the operation of an array of phonological processes, either vowel reduction (i.e., centralization of vowel’s formants and shortening of its duration, Lindblom, 1963) or consonant elision/assimilation/epenthesis, which occurs within and across a word boundary.

Processes occurring within words are relatively straightforward to explain as they occur within one lexical unit and have a relatively narrow domain. Cross-word phonological processes and ensuing reduction have been explored within the paradigm of the Production Planning Hypothesis (PPH, Wagner, 2012). Most theories (e.g., the variationist approach) seem to assume that all contextual information is available and retrieved simultaneously when a process transcends the boundaries of the word (Lange and Laganaro, 2014; Tamminga et al., 2016; Tamminga, 2018). PPH stipulates a more random way in which planning unfolds, departing from the linear assumption and leaning toward the view that only one word may be planned ahead at a time. PPH proposes that, apart from phonetic factors, phonological processes can be accounted for with a set of variables linked to production planning (called planning proxies), such as time pressure, speech rate, and degree of phonological/semantic/syntactic complexity of the following material. Other variables are probabilistic effects (Tanner et al., 2017; Kilbourn-Ceron et al., 2020) or duration of pause (Tanner et al., 2017).

The degree to which pronunciation of words may vary in colloquial speech due to phonological processes is impressive: Johnson (2004) reports that in spontaneously produced American English, over 60% of words deviate from their citation forms in at least one segment, whereas 6% of words miss at least one syllable in comparison with their full forms. For this reason, reduced forms may be potentially challenging for non-native speakers of English. Cruttenden (2008) observes that a second language is often learned on the basis of words in isolation and encourages EFL learners to familiarize themselves with assimilatory tendencies and weak forms. In a similar vein, Ernestus and Warner (2011) quote the form *yeshey* as a heavily reduced form of *yesterday*, stressing that reduced forms cannot be looked up in a dictionary by learners of English, nor can they be explained by native speakers who are usually not aware of reduction processes. Shockey (2003) points to a lack of significant contact with reduced forms when learners of a second language are taught by non-native speakers.

Apart from these problems, reduced forms related to consonants are governed by language-specific mechanisms, which add to difficulties in their production and perception. For instance, English and Polish (Dunaj, 1985, 2006; Madejowa, 1987, 1993; Madelska, 2005; Orzechowska, 2019; Zydorowicz, 2019) frequently exhibit the process of consonant cluster reduction (CCR), while in Greek, parallel CCR processes are rare. Shockey and Bond (2012) report that Greek learners of English find recognition of consonant cluster reduction challenging as they are not exposed to this particular variability in their L1. Polish learners of English, on the other hand, encounter fewer difficulties in recognizing English CCR processes. In a similar vein, a study by Shockey and Ćavar (2013) makes a number of phonotactics-related predictions, including those of Spanish, Latvian, and Greek learners of English.

### 1.1 Previous studies

Although the perception of reduced forms by native users of a language has been previously studied (e.g., Dilley and Pitt, 2010; Warner et al., 2012; Zimmerer and Reetz, 2014) and accounted for with usage-based and exemplar theories (e.g., Bybee, 2013), insights into the perception of casual speech by non-native speakers are infrequent in comparison. The vast majority of perception studies in language acquisition of casual speech, however, investigate vowel reduction, e.g., Smiljanic and Bradlow (2011), Sumner (2011), Van Dommelen and Hazan (2012), Ernestus et al. (2017), Brand and Ernestus (2018), and Morano et al. (2019). Thus, the area of consonant reduction appears to be underrepresented in research on reduced forms, with the notable exceptions of studies by Pearman (2004) and Shockey and Bond (2012).

The study by Pearman (2004) investigated the perception of casual English in L2 by Catalan learners, linking perception to the level of proficiency in English. Shockey and Bond (2012) tested the correct identification of reduction processes on Polish and Greek learners of English and concluded that the Polish learners outperformed the Greek ones. In particular, Pearman (2004) tested an array of reduction processes such as palatalization, place/manner assimilation, vowel/consonant weakening, and deletion. She gated the sentence *Is your friend the one that cannot go to bed by ten?* in 80 ms steps and presented it to 24 learners of English, both at the beginner and advanced levels of proficiency, and to a control group of 12 native speakers. She found that the outcomes of the advanced experimental group were consistently lower in comparison with the natives, and so were the scores of the beginner groups compared to the advanced one. Pearman (2004) also analyzed the level of confusion and concluded that with an increase in L2 experience, learners were perceptually readjusting in the direction of L2 phonological processing.

Similarly, Shockey and Bond (2012) used the gating technique in 50 ms gates on Greek and Polish learners of English. These groups were matched in terms of proficiency; thus, instead of proficiency level, language typology became a factor. To the authors’ surprise, Polish learners of English scored nearly as high as native speakers and far surpassed Greek learners in recognition of reduced forms. Shockey and Bond (2012) concluded that “[a] possible reason is that Poles have similar syllabic patterns in their own language, while Greek syllable structure is entirely different and, on the whole, simpler” (Shockey and Bond, 2012, p. 208). Shockey and Ćavar (2013) explained this finding further with language-specific differences, claiming that Greek learners of English are not accustomed to recognizing reduced forms affecting, e.g., consonant clusters, as Greek has few of them.

In discussing previous research on the perception of reduced forms by non-native listeners, a number of distinct blind spots can be identified. First, much less is known about the mechanisms governing perception of reduction processes affecting consonants than vowel reduction. Second, the two studies reported above use rather scarce input to test the perception of second language learners, i.e., one read sentence that contains a small selection of reduced forms and was produced by only one speaker: *Is your friend the one that cannot go to bed by ten?* [ɪ**z j**ə frend ðə wʌn ðə**t k**æn**t g**oʊ **t**ə bed baɪ ten] (Pearman, 2004) and *So it was quite good fun, actually, at the wedding, though…* [səʊ ɪ**t w**əs kwaɪ**t g**ʊ**d f**ʌn ˈækʃəli ə**t ð**ə ˈwedɪ**ŋ ð**əʊ] (Shockey and Bond, 2012). Third, the relatively low number of participants in the two studies, 24 in Pearman (2004) and 31 in Shockey and Bond (2012), prevents making generalizations about the perception of casual speech by learners and calls for more research. Fourth, the two studies lack the variability of tokens and speakers used in their experiments. In the spirit of corpus phonology (CP), the employment of a speech corpus seems a more suitable choice and would allow the drawing of more reliable generalizations than one sentence produced by one speaker. CP may be explained as “a novel methodological approach in phonology, denoting the use of purpose-built phonological corpora for studying speakers’ and listener’ knowledge and use of the sound system of their native language(s), the laws underlying such sound systems, L1 and L2 acquisition” (Gut and Voormann, 2014, p. 13).

To address these urgent neglects, a study was designed that seeks to fill the gap of using scarce input to investigate a wider array of consonant reduction processes and test a large number of tokens from a speech corpus on a significant number of subjects. The study reports the results on the perception of reduced forms in English by Polish learners of English and considers exclusively reduction processes affecting consonants: /t, d, h/ deletion, fricativization, assimilation of the place of articulation, and Yod coalescence. In particular, the study verifies the accuracy (referred to as Acc) and speed (reaction time, RT) of perception of reduced forms and makes a contribution to the area of perception of English casual speech by non-native users via the employment of a speech corpus.

Previous studies on native perception have typically addressed vowel reduction from the following angles: segmental context (Mitterer and Ernestus, 2006; Mitterer et al., 2008; Zimmerer and Reetz, 2014), word context (e.g., Van De Ven et al., 2011), word probability (van de Ven et al., 2012), speech rate (Dilley and Pitt, 2010), phonotactics (Spinelli and Gros-Balthazard, 2007), and syntax (e.g., Viebahn et al., 2015). As for the perception of vowel reduction by non-native listeners, the variables were as follows: high proficiency learners (Nouveau, 2012; ten Bosch et al., 2016; Wong et al., 2017), phonotactics (Shockey and Ćavar, 2013; Ernestus et al., 2017), speech styles (Smiljanic and Bradlow, 2011), voice onset time (Sumner, 2011), vowel formants and duration (Van Dommelen and Hazan, 2012), frequency of occurrence and exposure to a word (Brand and Ernestus, 2018), and word exemplars effects (Morano et al., 2019). Regarding the non-native perception of consonantal reduction, the following factors have been considered so far: the proficiency level of learners (Pearman, 2004), linguistic typological differences (Shockey and Bond, 2012; Shockey and Ćavar, 2013), and a stay abroad in an English-speaking country (Shockey and Bond, 2012).

Considering that the factors listed above were investigated mostly in native speech and concerned vowel reduction, the present study aims to advance our understanding of the non-native perception of consonantal reduction by conducting a corpus-based analysis. Due to the paucity of studies on learners’ perception of reduced forms relative to native speakers’ perception, the study analyzes non-native listeners’ performance with native speakers as a control group (Pearman, 2004; Shockey and Bond, 2012). Instead of replicating the well-documented variables governing perception, the study explores the effects of semantic context (Van De Ven et al., 2011), the effects of a process type whose impact is known from production studies (e.g., Holst and Nolan, 1995) but has not yet been tested in the perception of reduced forms. The study also tests the effect of music education, which might influence the perception of casual speech as it does aid the production of segments (e.g., Magne et al., 2006; Milovanov et al., 2010; Salcedo, 2010).

### 1.2 The effects of semantic context

A number of production studies found that learners often rely on the meaning of the semantic context (e.g., Van De Ven et al., 2011). The present study extends this claim to the perception of reduced forms and includes the effects of lexical context, understood as the presence or absence of words preceding and following the reduced form in question. It aims to establish whether the presence of context facilitates the perception of reduced forms, as opposed to the absence of context (e.g., *they should have do***n***e* **b***etter* vs. *do***n***e* **b***etter*, place assimilation of /n/ to /m/ in the vicinity of a bilabial sound), which in turn derives from claims that L2 learners require a lot more acoustic signal such as vowel formants and formant transitions for consonants and/or a greater portion of the word than native speakers. Learners often need to hear the beginning of the next word before correctly identifying an item; in comparison, native speakers usually recognize the word before its offset (Nooteboom and Truin, 1980; Koster, 1987).

### 1.3 The effects of process type

A wide range of phonological processes is responsible for the difference between citation and reduced forms. This variety is language-specific and largely depends on the phonemic inventory of a language, word stress rules, and phonotactic constraints. Processes themselves operate in three ways: they can add a sound (*ham(p)ster*), delete it (*min**d** the gap*), or assimilate two or more sounds (*coul**d b**e*). Consequently, a process can belong to one of three major process groups: insertion, elision, and assimilation. Since they exert different phonetic effects, phonological accounts distinguish between categorical (e.g., Clements, 1985; McCarthy, 1988) and gradient types of processes (Browman and Goldstein, 1990; Barry, 1992). Assimilatory processes illustrate the gradient type as a change from sound A to sound B, which may involve intermediate stages, be incomplete, and leave a phonetic trace. Previous studies report that, e.g., an alveolar stop assimilating to a following labial or velar does show acoustic (Gow, 2003) or articulatory (Ohala, 1990; Nolan, 1992; Zsiga, 1995; Ellis and Hardcastle, 2002) traces of both alveolar and labial/velar place of articulation. We also know that extreme assimilation (i.e., complete blending of two places of articulation) results in considerable processing difficulties (Holst and Nolan, 1995). Categorical type of processes such as elision, on the other hand, neither leave a trace nor change one sound into another. Instead, they involve a complete realization. Studies on L1 production (Wright and Kerswill, 1989; Ellis and Hardcastle, 2002) and L1 perception (Hanique et al., 2013) attest to different effects of gradient and categorical processes, respectively. In this connection, the current study aims to explore the effects of process type on non-native perception. The idea that gradient processes have a different effect on learners than the categorical one seems worth pursuing.

### 1.4 The effects of musical background

The study investigates the effects of musical education, formal or informal (including singing), which may affect the perception of reduced forms. The claim that musical aptitude and linguistic skills are interconnected is well evidenced (e.g., Mithen, 2005; Jackendoff, 2009; Harvey, 2017), especially in the area of rhythm (Patel, 2003; Patel and Daniele, 2003; Mora and Gant, 2016). It has also been assumed that learners with musical talent and training achieve better results in pronunciation than learners without a musical background (e.g., Magne et al., 2006; Pastuszek-Lipińska, 2009; Milovanov et al., 2010; Salcedo, 2010). In the light of emerging evidence, music aptitude in SLA positively affects the production of certain aspects of English-connected speech: rhythm, elision, assimilation, and linking (Milovanov, 2009; Besson et al., 2011; Gordon et al., 2011; Balčytytė-Kurtinienė, 2018). Casual speech mostly consists of vowel reduction, specific alternation of stressed and unstressed syllables, and an abundance of weak forms and weak syllables; the study by Gordon et al. (2011) examined the perception of weak and strong syllables in songs, pointing to the role of songs in increased attention of listeners when the beat was aligned with a strong syllable. In the study by Besson et al. (2011), musicians and non-musicians were compared in their perception performance of pitch, vowel duration, and metric processing in casual speech by training transfer and demonstrated overall facilitation for musicians. As far as rhythm and duration of vowels are concerned, the study by Milovanov (2009) proved that learners with greater musical aptitude had better scores in recognition and discrimination tasks than learners with no such skills. Following these suggestions, the current study tests this claim in the perception of consonantal reduction in casual speech. The choice of this particular effect was also motivated by the willingness to incorporate one extralinguistic factor in the study, in addition to the two linguistic ones (i.e., the effects of lexical context and process type), since previous studies on native perception of reduced forms and their variants examine speech rate as an extralinguistic factor (Dilley and Pitt, 2010).

To sum up, the present study analyzes the perception of reduced forms by learners of English to test the effects of (i) lexical context, (ii) two types of reduced forms (categorical and gradient), and (iii) musical background. The second aim determines the choice of phonological processes as they need to represent both gradient and categorical types: /t, d, h/ deletion, fricativization, assimilation of the place of articulation, and Yod coalescence. The study also aims to compare the learner’s results to those of a control group of native speakers, and it was designed to use authentic, spoken English from a speech corpus in an SLA study.

### 1.5 The hypotheses

Pursuing the above aims, the study verifies three hypotheses:

Hypothesis 1 concerns the effects of lexical context on the perception of reduced forms and predicts that reduced forms with context are recognized more accurately and faster than reduced forms without context by learners, as reported in previous works (Nooteboom and Truin, 1980; Koster, 1987; van de Ven et al., 2012).
Hypothesis 2 addresses the effects of a process type. It is not unreasonable to expect that L2 listeners have a sharper perception of words with a missing sound (categorical processes) than for the potentially confusing effects of gradient processes such as place assimilation (e.g., change from /d/ to /g/), Yod coalescence (replacing /t, d, s, z/ in the vicinity of /j/ with / ʃ, ʒ, tʃ, dʒ /), or fricativization (/h/−like sound instead of a stop), which might cause perceptual difficulties. Thus, hypothesis 2 assumes that gradient reduction (fricativization, place assimilation, and Yod coalescence) is perceived slower and less accurately than the categorical one (/t, d, h/ deletion; Holst and Nolan, 1995; Hanique et al., 2013).
Hypothesis 3 extends the role of musical background from production and perception of individual sounds and suprasegmentals to casual speech (e.g., Besson et al., 2011). It stipulates that learners with musical backgrounds perceive reduced forms more accurately and in a shorter time than those without them, testing the effect of music skills or education on non-native perception.

## 2 Materials and methods

### 2.1 Subjects

One hundred and two adult Polish learners of English in the full- and part-time English and Russian-English programs at Adam Mickiewicz University participated in the study. Ninety-nine subjects ranged in age from 18 to 24 years, with a mean age of 21 years. The remaining three participants were aged 17, 37, and 40 years. All of them were native speakers of Polish at an English proficiency level, which can be described as advanced. In particular, the subjects were continuing their education in the second year of their postgraduate studies. Following their first year, they had to take an exam, placing them at advanced level described as B2+ (University of Adam Mickiewicz, n.d.). For this reason, the study cannot include proficiency level due to the highly even level of English among the subjects considered: they all were advanced students of English.

In addition, the participants had a similar background in phonetics since all of them had attended two obligatory courses in pronunciation of English: an EFL course in pronunciation and a course in phonetics and phonology. The former consists of drilling vowels, diphthongs, and consonants with the use of a multimedia program (Sawala et al., 2024). The latter is of a theoretical nature, introducing concepts such as phonemes, phonotactics, phonostylistics (including reduction processes), and phonological theories.

To tease apart the effects of context in the perception of reduced forms, the participants were assigned to one of two groups: 52 subjects listened to reduced forms without context (the NoContext group), whereas the remaining 50 participants constituted the Context group, which heard the same reduced forms as the NoContext group, but in context. A control group of 14 native English listeners served to demonstrate that the L2 learners were affected by processes of connected speech and not some other factors that might be present in the signal. Thus, 8 native listeners were presented with the stimuli in context, and 6 native speakers of English listened to the stimuli without lexical context. Table 1 presents their accents.

**Table 1:** Native speakers’ places of residence.

Control group for the NoContext group		Control group for the Context group	
Initials	Place of residence (by 18)	Initials	Place of residence (by 18)
PN	UK (Manchester)	DL	UK (Solihull)
TA (a)	US (California, Oklahoma, Ohio)	SN	Canada (Alberta)
BF	UK (Leeds, Hull)	TdP	Australia (Western Australia)
TA (b)	Ireland	RH	Canada (British Columbia)
SB	UK (Norfolk, Essex)	CP	UK (South)
CW	UK (Oldham)	SH	UK (North Yorkshire)
		BN	US (California)
		RJ	US (Oregon)

One may wonder whether accent variability, reported in Table 1, might exert an influence on the comparison between native and non-native listeners. With the exception of fricativization being reported mostly for the UK dialects (Lodge, 1984; Beal, 2010), all remaining reduction processes (h, t, d-deletion, assimilation of place, and Yod coalescence) occur in English regardless of the dialectal differences (Wells, 1982; Kortmann and Upton, 2008). For instance, h-dropping is a function of grammar (e.g., unstressed personal pronouns), and place assimilation is conditioned by the phonetic context and is not specific to a variety. As a textbook variable of sociolinguistic variation, /t, d/ deletion has been investigated in a number of varieties, such as American English in general (Neu, 1980; Guy, 1992; Guy and Boberg, 1997), in particular Philadelphia English (Guy, 1980), New York English (Labov et al., 1968), Appalachian English (Hazen, 2011), African American Vernacular English (Wolfram, 1969; Fasold, 1972), and British English (Tanner et al., 2017), specifically York English (Tagliamonte and Temple, 2005).

In addition, the study examines an extralinguistic factor related to the subject background, i.e., musical background by which the study understands the ability to play an instrument, either self-taught or obtained via formal music education and/or singing in a choir or a band. Musical background was established using a questionnaire, and results are reported as follows: 27 subjects from NoContext Group (52%) had musical background. However, only for 6 participants (22%) was the education formal. The duration of years spent in musical school ranged from 4 to 7 years and referred to the first degree (5.5. years on average). In Poland, there are three types of musical schools: first-degree and second-degree musical schools, which require 4 to 6 years of training, and third-degree (School of Music, 6 years) musical schools. In comparison with informal musical education (e.g., private lessons), the musical school offers an intensive program (courses at least twice a week), including theoretical modules such as rhythm, harmony, and composition. In the Context Group, 36% (18 participants) claimed to have some musical background. Within the group, 66% (10 subjects) received a formal musical education, both the first and second degrees. Duration of schooling ranged from 2 to 9 years, with an average of 5.4 years.

To sum this subsection up, the study tests the perception of reduced forms in English in the following experimental setting: the analysis is performed on two groups of subjects (learners and native users of English) and aims to tease apart the effects of lexical context (hence, four groups: NoContext leaners, NoContext native speakers, Context learners, and Context native speakers) as well as the effects of musical background in perception (for learners only, four groups: NoContext group musical background, NoContext group no musical background, Context group musical background, and Context group no musical background).

### 2.2 Speech material

The study was corpus-based and used reduced forms from the Phonologie de l’Anglais Contemporain corpus (PAC, Durand and Pukli, 2004). It contains recordings of 9 female speakers of Lancashire, collected between 2001 and 2002. PAC’s structure is as follows: a list of words, a read passage, and formal and informal interviews. Both interviews were loosely structured and conducted in an informal setting, at informants’ homes or workplaces. The formal interview was conducted by a French speaker of English, a stranger to the informants. The informal interview, on the other hand, was carried out by a native speaker of Lancashire who was either a relative or a friend (or a family friend) of the informants. Due to these differences, topics in the formal interview covered past events, memories from school, family situations, jobs, and travels abroad. The informal part concerned current topics such as housing problems, plans for Christmas, and gossip about common friends, relatives, and neighbors. For this study, the speech material comes from informal interviews to ensure fully casual speech. Although the choice of the English accent in this study may seem somewhat arbitrary, one particular argument speaks in favor of PAC: the PAC corpus of Lancashire was deemed appropriate since it has already been annotated and exploited with respect to reduced forms. Other corpora of spoken English are much less easy to mine as none of them are annotated beyond segments.

With regard to stimuli, particular care was taken to select simple words and phrases in which a reduction process occurred either within a word or across word boundaries. Lexical items used to test reduction were nearly all high-frequency items (following the British National Corpus, 2017, for frequency rankings, *cf.* Appendix 1), which excludes the possibility that the subject did not understand the word(s) they were supposed to identify in reduced forms.

The study’s author manually cut the stimuli from the PAC corpus using Audacity software and exercised caution to include, e.g., the release stage of a stop in the signal. The stimuli, fed in the WAV format into an E-prime script, were high-quality recordings in the .wav format. In total, the study tested the perception of 287 stimuli, representing six tested processes: /t/ deletion, /d/ deletion, /h/ deletion, fricativization, place assimilation, and Yod coalescence. The NoContext group listened to 170 reduced forms without context (30 per each of six categories with the exception of fricativization, totaling 20 instances since most of the examples of fricativization in the corpus were preglottalized), whereas the Context Group heard 117 stimuli (20 per each process and 17 for fricativization) with context; the stimuli with lexical context were longer, and for this reason, their number was lower. It has to be clarified that context does not entail a full sentence, as sentences in casual speech are frequently long, complex, unfinished, or interrupted. Instead, a smaller portion of an intonation phrase within a breath group and defined by pauses were selected by hand, ranging from one to 15 following and preceding words. All stimuli were presented in random order for every subject. Table 2 exemplifies the stimuli; for a full list, see Appendices 1, 2.

**Table 2:** Examples of stimuli.

Process	Stimuli without context	Stimuli with context
/t/ deletion	Mus**t** be	They mus**t** be altered
/d/ deletion	Poun**d**s	Nearly 2,000 poun**d**s
/h/ deletion	See**h**er	I still see**h**er occasionally
Fricativization	O**b**viously	O**b**viously I had a family to look after
Place assimilation	A**nk**le	Then I’ve sprained my a**nk**le
Yod coalescence	The**s**e**y**ears	But you have remembered it all the**s**e**y**ears

### 2.3 The procedure

**Figure 1:**
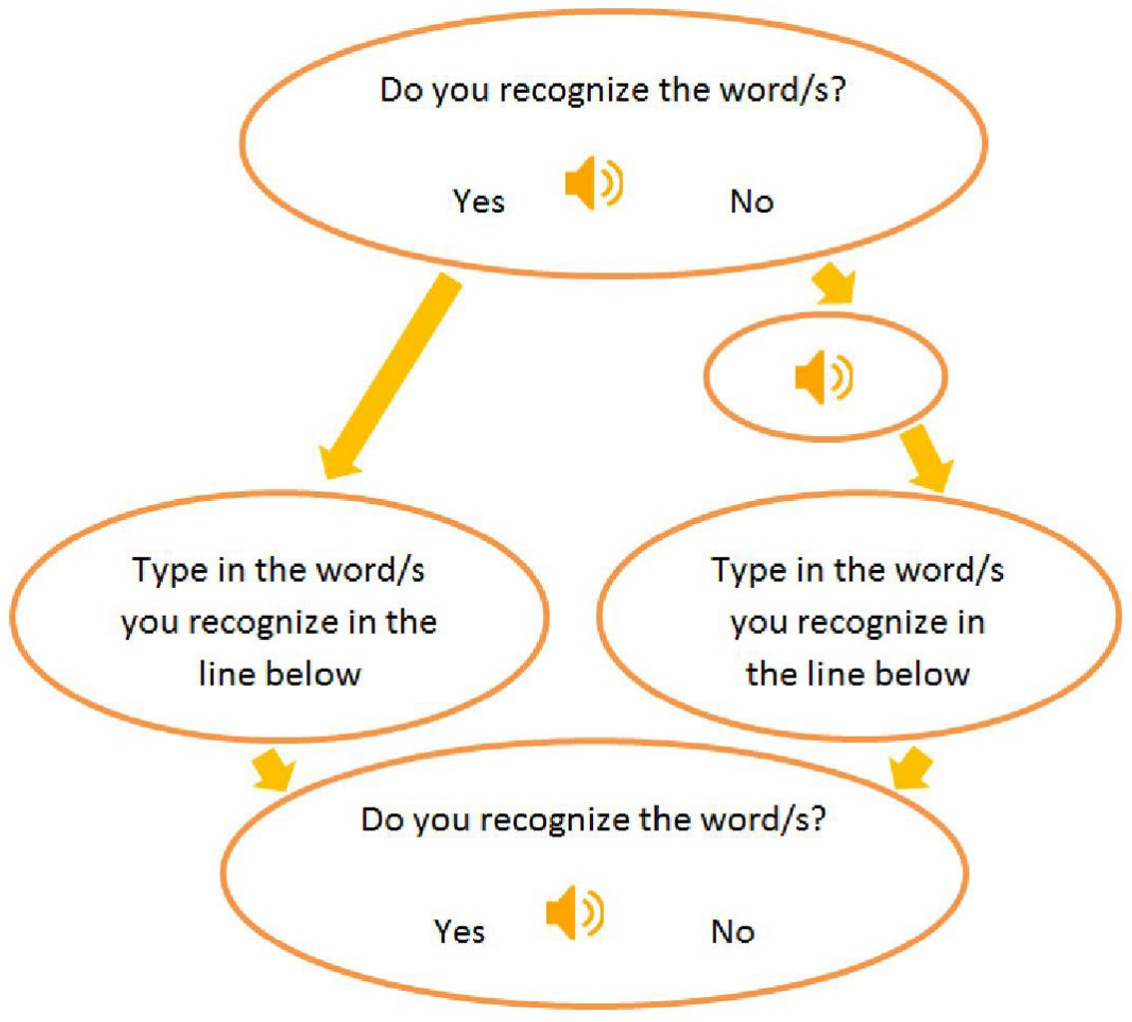
The E-prime task.

Participants completed the experiment in the Language and Communication Laboratory at a Polish state university in a single session. Before the study, they filled in a questionnaire that furnished metadata on the subjects and their language history. The questionnaire included sections about the subjects’ musical backgrounds. Next, the subjects proceeded to perform the experiment in E-prime 2.0, equipped with Sennheiser headphones. The script in E-prime consisted of a trial session, a 2-min-long recording which introduced the subjects to the Lancashire dialect, speakers, and the task. The task contained no visual elements except for the icons of a loudspeaker to indicate a sound file. The instruction was the same for both groups, and the subjects were asked whether they recognized the word/s they heard (measuring reaction time) and to type in the words they recognized (to capture accuracy). Failure to provide a Yes answer indicated a lack of understanding of a stimulus and resulted in hearing the stimulus once more; each stimulus could be played twice. Only then did the next screen appear with a box to type. The task is visualized in Figure 1.

The subjects were not aware of the purpose of the study; they only knew that they were going to listen to a few samples of Lancashire. Participation was voluntary and took place before or after their regular courses.

The study analyzed a total of 14,690 answers provided by Polish learners of English, as well as 1956 answers from native English speakers. Although the E-prime script had automatically assigned points for correct answers, the study’s author ran an independent, manual analysis of the obtained results to include answers with typos or orthographical errors. The analysis was binary: a point was assigned if the answer demonstrated that the subject had correctly understood the reduced form in question (even if the rest of the token was not understood correctly), and zero points if there was a failure to comprehend the meaning of the reduced form. That involved cases where L2 learners typed correctly a word or two words affected by a process and the preceding and upcoming words were not understood.

The measurements of RTs were triggered when the subjects pressed the Yes/No button. In particular, RTs were measured for both No and Yes answers by default. In the course of analysis, the results from the No column in the spreadsheet generated by the script were not taken into account. Following this step, I also performed a manual verification of the answers in the Yes column, where the subjects were asked to write what they had heard. The study’s participants were not specifically instructed to provide the IPA type of transcription of the words they heard and supposedly recognized. Some subjects attempted some sort of impressionistic transcription, and others used an orthographic one. It has turned out that certain subjects prescriptively corrected a reduced form, whereas other participants managed to capture the effects of a process. For these reasons, I had to read every single typed response and decide whether the words were recognized correctly as well as exclude the” mondegreens” mishearings. For instance, *makes you* was frequently written down as *makes sure*. The final step of that manual verification was another column in the spreadsheet annotating if this response (and the RT) could be considered in the analysis. Categorical/gradient reduction was measured in the same way as overall word recognition, by hand, based on the typed response with filtered deletion or non-deleting processes.

One-way ANOVA was run to assess the differences between accuracy and reaction time between various groups, i.e., Context and NoContext groups, native speakers and learners, gradient and categorical type of processes, and musical backgrounds. Next, the effect size is reported as Cohen’s *d* for equal groups and slightly modified for comparison between native and non-native listeners due to the difference in sample size with Hedges’ *g*. Two-way ANOVA (without replication) was run to assess the difference between subjects with and without music education since there were two independent variables, i.e., musical background (Hypothesis 2) and lexical context (Hypothesis 1).

## 3 Results

### 3.1 Results for hypothesis 1

**Figure 2:**
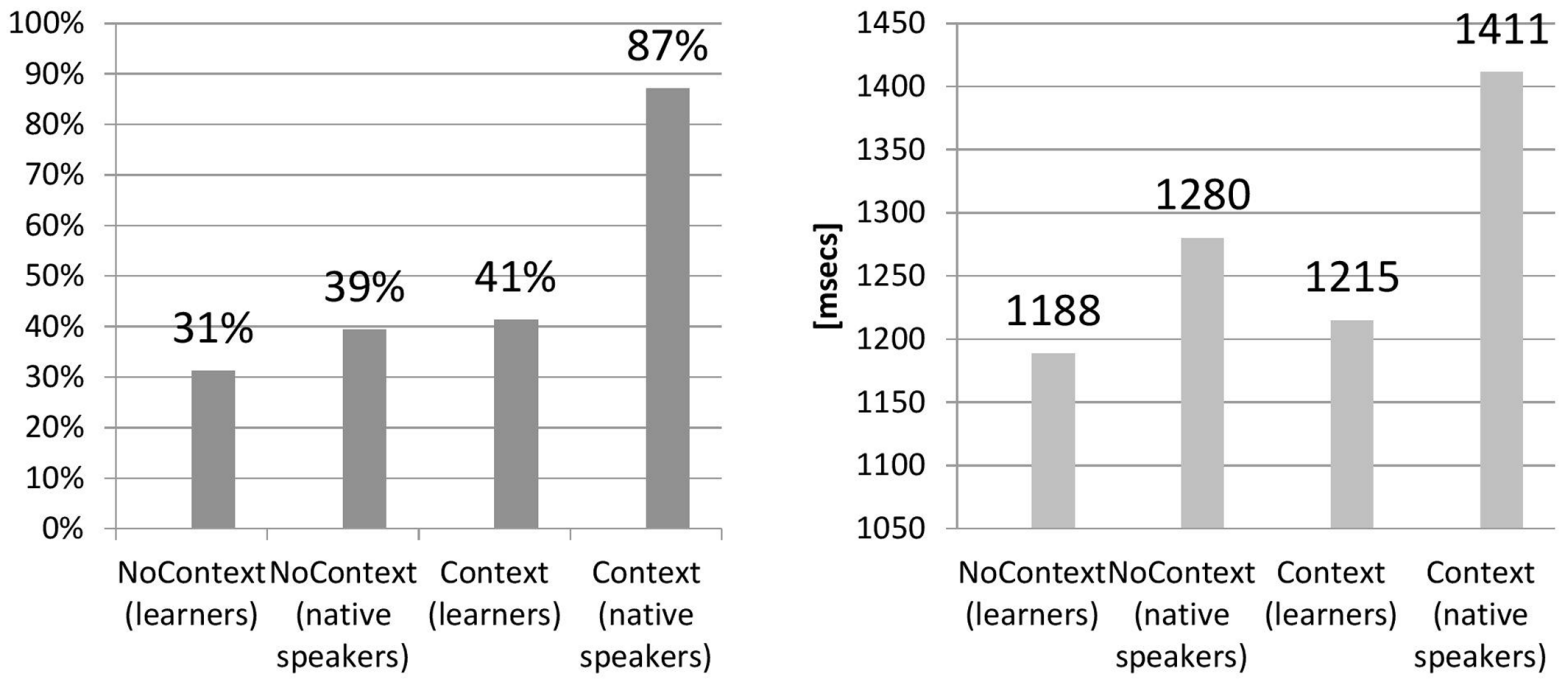
Comparison of Acc and RT between groups.

The first hypothesis predicted that the presence of lexical context boosts the perception of reduced forms. Figure 2 presents the results.

Regarding accuracy, although a difference between learner’s Context and NoContext groups was observed, *F*(1,100) = 52.76, *p* < 0.05 (NoContext: M = 53.25, SD = 10.56, *N* = 52; Context group: M = 48.42, SD = 12.01, *N* = 50), the value of Cohen’s effect size for the differences between learners’ groups (*r = 0.21, d* = 0.43) suggested small practical significance between the context and no-context conditions (31% and 41%, respectively). Concerning the comparison between learners (NoContext: M = 53.25, SD = 10.56, *N* = 52, Context: M = 48.24, SD: 12.01, *N* = 50) and native speakers (NoContext: M = 67, SD = 12.02, *N* = 6; Context: M = 102, SD = 7.97, *N* = 8), there was a difference for the NoContext Group, *F*(1,75) = 94.58, *p* < 0.05, as well for the Context Group, *F*(1,75) = 1720.88, *p* < 0.05. Again, contrary to the results of a mere comparison, it turns out that the effect size for the NoContext learners vs. NoContext native speakers groups was weakly significant (*r =* 0.34*, d* = −1.18, *g = −1.26*). It was highly significant, *d = −*5.26*, r =* 0.88*, g* = −4.56, only for the Context groups of learners and native speakers. In the analysis, the strength of the association (*r*) was calculated using *d* for unequal groups.

The variation of the effect size in the Context vs. NoContext conditions merits further analysis, which reports exact differences between the groups of learners and native speakers. To this end, Tukey’s Honest Significant Difference test (HSD) was run as a post-hoc test. A Tukey’s *post hoc* test revealed that with an error rate of 0.05, there was no statistically significant difference between the learners’ Context and NoContext groups as their confidence interval contained zero (difference points = −4.83, *p* = 0.14), unlike for native speakers (difference points = 35, *p* = 0.00). The post-hoc test also demonstrated that the improvement in the performance was significantly lower for the learner vs. native speakers groups in the NoContext condition (difference points = 13.75, *p* = 0.03) compared to the difference between learners and native speakers in the Context condition (difference points = 53.58, *p* = 0.00). An interesting observation is that the difference between learners and native speakers in the NoContext condition was statistically significant. This implies that native users of English outperformed the learners even if the lexical context was missing.

Figure 2 also suggests between-groups differences for reaction time, which were then investigated with one-way ANOVA. The compared groups of learners differ significantly, *F*(1,100) = 420.27, *p* < 0.05, allowing us to observe that the Context Group (M = 1359.31 ms, SD = 786.17, *N* = 50) recognized reduced forms faster than the NoContext Group (M = 1466.27 ms, SD = 605.21, *N* = 52). In comparison with native speakers, tokens without context were recognized slower by learners than by native speakers, *F*(1,75) = 314.26, *p* < 0.05 (learners: M = 1466.27 ms, SD = 605.21, *N* = 52; native speakers, M = 1284.24, SD = 179.36, *N* = 6). The same tendency was observed for the Context Group: *F*(1,75) = 24.95, *p* < 0.05 (learners: M = 1359.31 ms, SD = 786.17, *N* = 50; native speakers, M = 1411.28, SD = 469.29, *N* = 8). The effect size between two non-native speakers groups was not significant: *r = −*0.07, *d = −*0.14. With regard to the comparison of NoContext and Context between native speakers and learners, the effect size failed to reach any significance: *r* = 0.13, *d* = 0.41, *g* = 0.31 and *r* = 0.03, *d* = −0.08, *g* = −0.07, respectively.

Having applied a post-hoc test, Figure 2 can now be correctly interpreted. Thus, the study verifies Hypothesis 1 as follows: learners tested in the Context group recognized reduced forms faster but not more accurately than learners in the NoContext group. This outcome strongly suggests that the inclusion of semantic context did not help learners correctly identify reduced forms. In contrast, within the group of native speakers, semantic context did not shorten their reaction time but significantly improved their performance in comparison with the no-context group. Comparing the performance of native vs. non-native groups, native speakers have considerably outperformed learners in the perception of reduced forms, both with context and without context. Overall, hypothesis 1 was positively verified for native speakers of English and negatively for learners.

Following statistical analysis in terms of Cohen’s effect size and *post hoc* Tukey HSD test, the outcomes obtained for hypothesis 1 reveal a significant difference between L1 and L2 users, which merits further investigation. In an attempt to cast light on this difference, the study explores two alternative suggestions that might account for the poor performance of Polish learners: the length of reduced form (calculated as phone density) and the influence of L1. These two possible explanations are discussed in turn.

Hypothesis 1, pertaining to the effects of lexical context, brought an interesting result for the learner group: even though the presence of context did not significantly aid accuracy in comparison with native users of English, the reaction time of learners was shorter than that of native speakers (Figure 2). This raises the question about the role of cognitive load since the tokens varied greatly in length due to the presence or absence of context. Tokens without context were consequently considerably shorter than the ones with context; still, there were differences between the two as the tokens with context were extracted from the corpus along natural boundaries constituted by intonation contours as well as by semantic/syntactic units and as a result were not of equal length. Given the limitations of working memory and increased cognitive load associated with longer phrases for learners, it can be expected that the length of a token has an influence on reaction time and, perhaps, accuracy in perception. In this study, semantic context or its lack determined the length of a token. As a follow-up on Hypothesis 1, the study now attempts to establish the influence of token length on perception operationalized as phone density and analyzed across low-, mid-, and high-density groups. Phone density was calculated as the number of phonemes in the phrase or sentence that contained a reduced form. For the NoContext group, it denoted the total number of phonemes in the token. Tokens with context were significantly longer; consequently, the sum of phonemes preceding and following the reduced form (but excluding the form itself) constituted phone density for the Context Group. The rationale behind this relied on the assumption that low density triggers higher accuracy and shorter reaction time (and that high density hinders correct perception and reaction time) and consists of correlating accuracy with reaction time for correct answers with three groups: tokens of low, mid, and high phone density.

The numbers for density groups (high, mid, and low) represent the actual numbers of phones in the phrases from the stimuli. In selecting the stimuli for the study, I used an intonation phrase or a pause as a unit/boundary so that the context would make a discourse unit as well. The criteria for assigning the number of phones to a particular group were as follows: the groups should not overlap nor be too close, and they should make generalizations possible.

**Figure 3:**
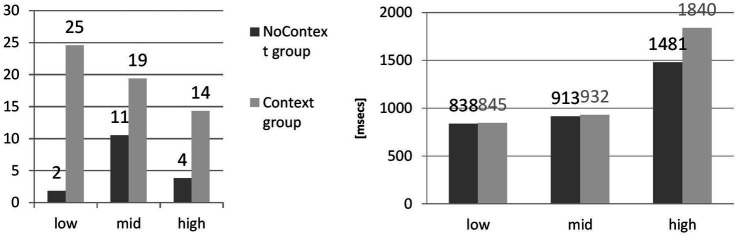
Correct answers across density groups (accuracy, unit: number of phonemes) and reaction time.

Table 3 presents density groups, whereas Figure 3 summarizes the results. The results represent the means of correct answers per each density group (low, mid, and high) as well as means of accuracy and reaction times across density groups for learners.

**Table 3:** Phone density across groups.

NoContext	Number of neighboring phones	Example	Context	Number of neighboring phones	Examples
3–4	Low	*I had*	3–5	Low	*Did you get her*
7	Mid	*Last year*	15–16	Mid	*I still see her occasionally*
10–11	High	*childminder*	25–39	High	*I could not turn him on my own that is why I always had to ask for help*

The results for Context Group (low-density: M = 25, SD = 15, *N* = 9; mid-density: M = 19, SD = 14, *N* = 10; high-density: M = 14, SD = 11, *N* = 10) display the tendency toward better recognition of reduced forms in shorter tokens; for NoContext (low-density: M = 2, SD = 1, *N* = 7; mid-density: M = 11, SD = 7, *N* = 6; high-density: M = 4, SD = 7, *N* = 7), mid-density seems to boost perception. The results are statistically significant (NoContext Group: *F*(2,17) =33.86, *p* < 0.05; Context Group: *F*(2,26) =21.61, *p* < 0.05).

Reaction time neatly dovetails with the number of phonemes in the tokens: the longer the stimuli, the more time it took for learners to process a reduced form [(NoContext Group: *F*(2,17) = 909.62, *p* < 0.05; Context Group: *F*(2,26) =3840.74, *p* < 0.05). No Context: (low-density: M = 838, SD = 455, *N* = 7; mid-density: M = 913, SD = 479, *N* = 6; high-density: M = 1,481, SD = 1,797, *N* = 7) and Context (low-density: M = 845, SD = 220, *N* = 9; mid-density: M = 932, SD = 268, *N* = 10; high-density: M = 1,840, SD = 1,770, *N* = 10)].

The length of a reduced form had an impact on perception only in the Context group (Figure 3), and this has been trending in the expected direction: the longer the phrase, the more difficult identification was. For the NoContext group, on the other hand, the role of phone density is not so clear, as low and high density produced similarly low results. Perhaps the group without lexical context was insensitive to the length of the form, and the mild effect of mid-phone density is purely accidental as it does not quite follow the pattern evidenced for the Context group. Nevertheless, it seems that the phone density account only partly predicts the perception of reduced forms by learners of English. Overall, the analysis of phone density as a possible predictor of perception has confirmed the intuitive connection between phone density and reaction time in the learner’s group: the shorter the token, the shorter the reaction time, regardless of the presence or absence of context. Regarding accuracy, the same relationship was observed for the context group: the longer the token, the worse the perception. The NoContext group, however, does not follow this tendency. It seems that neither low nor high phone density facilitated the correct identification of a token. Instead, mid-density was most favorable for perception.

The outcomes for token duration (expressed as phone density) provide some explanation for the effects of context, at least for reaction time; the other way to shed more light on non-native perception is to consider typological differences between L1 and L2 as it is very likely that typology plays a role. Out of six casual speech processes present in English (/t, d, h/ deletion, fricativization, place assimilation, and Yod coalescence), Polish has only two: /t/ deletion and assimilation of place (and of manner and voicing, Wierzchowska, 1980; Sawicka, 1995; Madelska, 2005). One may assume that the processes, which both languages have in common, will be the most salient for learners’ perception (Table 4).

**Table 4:** Typological differences between Polish and English.

Process	Polish	English
Fricativization	−	+
Yod coalescence	−	+
Assimilation of place	+	+
h-deletion	−	+
d-deletion	−	+
t-deletion	+	+

**Figure 4:**
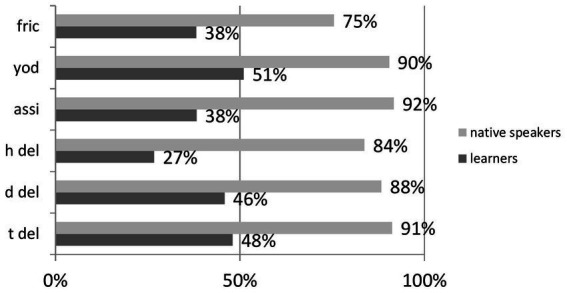
Distribution of accuracy across groups of reduced forms.

Below is an illustration of the correct perception of reduced forms across reduction categories in the form of Figure 4 (shown for the ContextGroup only since the NoContext group exhibited poor perception in general).

Regarding descriptive statistics, M = 403,5 SD = 95.46 for learners, and M = 574, SD = 143.68 for native speakers. Figure 4 reveals that for /h/ deletion, a process that is absent from Polish phonology (Table 4), perception was at the lowest level of 27%. The outcomes for fricativization (not present in Polish) and place assimilation (present in Polish) are the same, i.e., 38% of correct recognition, yielding mixed results in explaining perception with typological differences. /t/ deletion, another reduction process common in L1 and L2, was identified on nearly the same level of accuracy as /d/ deletion (not present in Polish) and less accurately than Yod coalescence (also not present in Polish). Should typological differences matter, we would expect place assimilation and /t/ deletion to rank the highest in terms of correct identification, followed by phonological processes not known in L1. Figure 4 clearly demonstrates that this, however, was not the case and undermines the influence of L1 on L2 in the area of reduced forms.

### 3.2 Results for hypothesis 2

**Figure 5:**
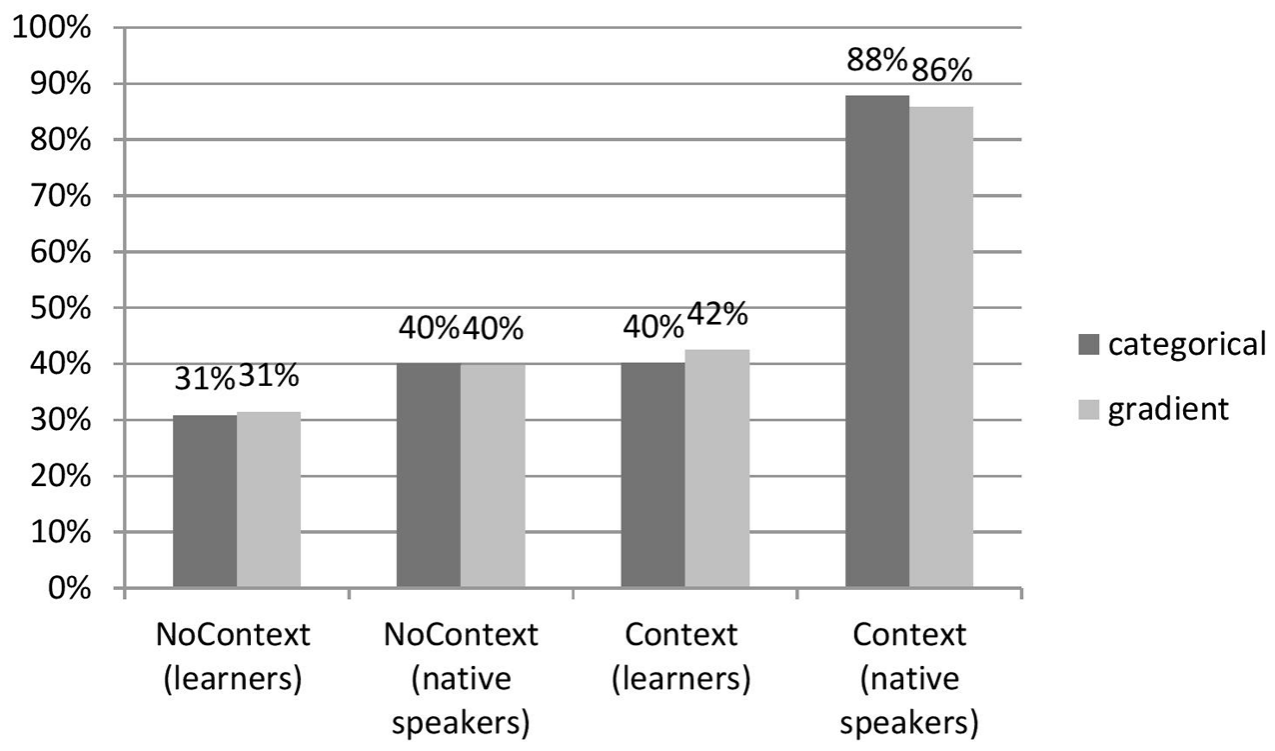
Distribution of accuracy within reduction categories across groups.

Hypothesis 2 predicted that the subjects perceive words affected by processes of categorical reduction more accurately than the gradient ones. This prediction was made on the grounds that categorical reduction deletes a sound without an acoustic trace, and the difference between citation and reduced form is consequently more perceptually salient than a change of one sound’s place on articulation into another (gradient reduction). Figure 5 displays the outcomes of the analysis.

Raw percentages from Figure 5 show that the perception of gradient and categorical reduction was barely different for the two groups across two conditions. Nevertheless, the difference estimated by one-way ANOVA between groups trended in various directions. For categorical reduction, they are reported as follows: NoContext Group learners, *F*(1,101) = 70628.00, *p* < 0.05, M = 480.67, SD = 190, *N* = 4,680, and Context Group learners: *F*(1,4) = 0.006, *p* > 0.05, M = 401.67, SD = 117.14, *N* = 3,000. However, the effect size was very weakly significant, *r* = 0.24, *d* = 0.5, for the NoContext vs. Context Condition. Concerning gradient reduction, this is how effect size looked for learners: the NoContext group: *F*(1,101) = 334490.58, *p* < 0.05, *r = 0.46*, *d = 1.04* (the NoContext group: M = 442.33, SD = 147.29, *N* = 4,160; the Context Group: M = 259.40, SD = 201.17, *N* = 2,850). Thus, the effect size points to a medium (bordering weak) difference in gradient reduction for Polish learners of English.

The difference for native speakers was observed, *F*(1,101) = 784188.32, *p* < 0.05, for categorical reduction (NoContext: M = 624, SD = 222.14, *N* = 4,680, Context: M = 877.78, SD = 37.81, *N* = 3,000). The effect size, *r = −0.62*, *d = −1.59*, pointed to a medium-strong influence of the context on recognition. Regarding gradient reduction, the effect of a process type was robust, with *r* = −0.78, d = −2.50*, F*(1,101) = 1567800.14, *p* < 0.05 (NoContext: M = 537.33, SD = 39.72, *N* = 4,160, Context: M = 820.83, SD = 155.29, *N* = 2,850).

Following the effect size of the difference between categorical and gradient reduction (rather than mere values from Figure 5), the study reports that it was very weak in the no-context condition and weak in the context condition within the learners’ group, yielding the effects statistically insignificant. Within the group of native users of English, on the other hand, the difference between categorical (medium strong) and gradient (strong) reduction shows that the prediction of Hypothesis 2 was correct. Native speakers perceived categorical reduction more accurately than the gradient one. Similar to Hypothesis 1, Hypothesis 2 is thus corroborated for native speakers and rejected for learners.

### 3.3 Results for hypothesis 3

**Figure 6:**
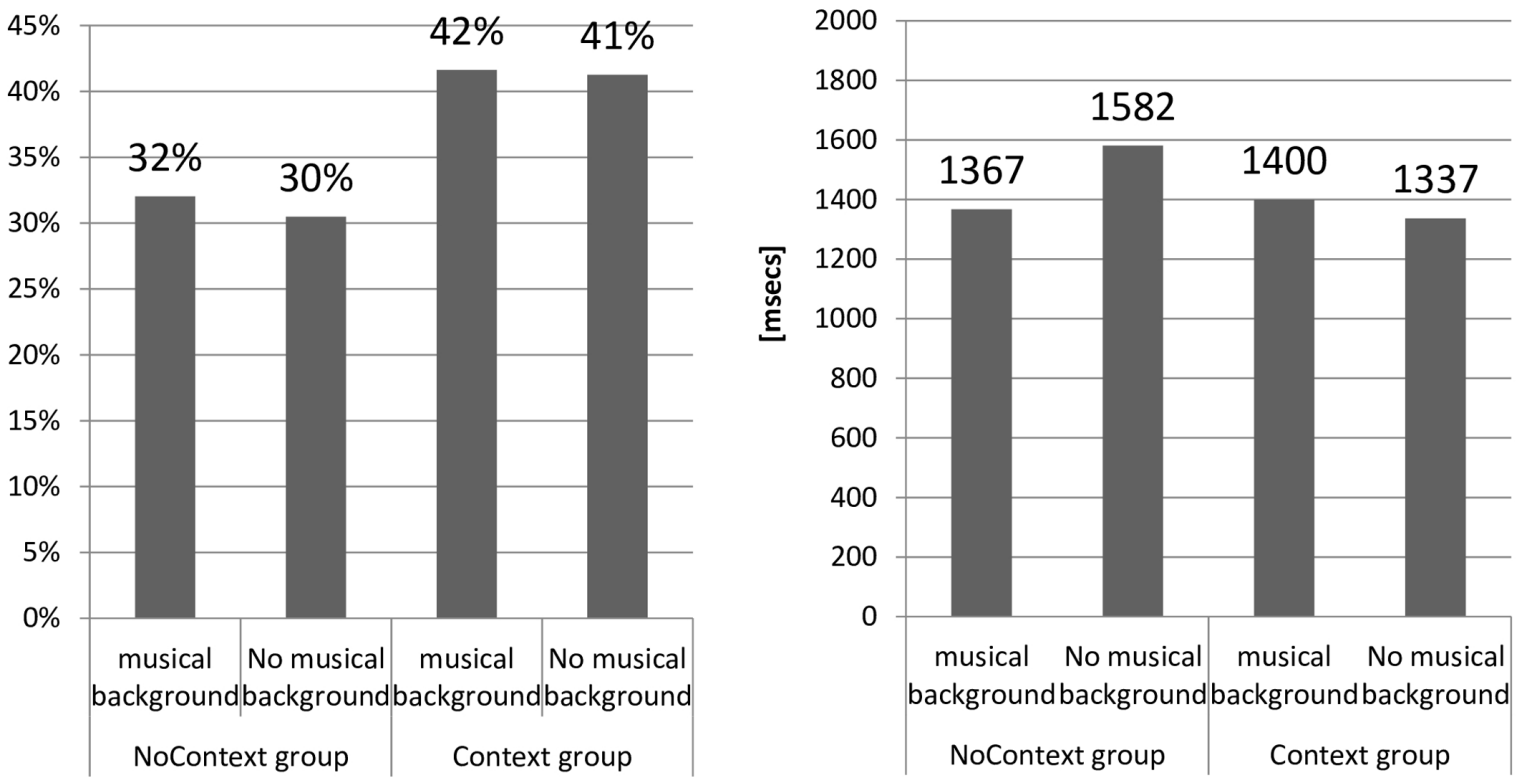
Acc and RT according to musical background.

The study hypothesized that learners with musical backgrounds perceive reduced forms better than those without. Figure 6 visualizes the obtained results.

Two-way ANOVA compared two groups of learners (learners with musical background, learners without musical background) in two conditions (with context and without context), revealing no difference in accuracy: for treatment (music), *F*(1,101) = 3.96, *p* = 0.19, and for other factors (context), *F*(2,101) = 50.52, *p* = 0.02; NoContext Group musical background, M = 37.48, SD = 7.13, *N* = 27, NoContext group no musical background, M = 35.67, SD = 7.45, *N* = 25, Context Group musical background: M = 48.70, SD = 14.14, *N* = 18, and Context group no musical background: M = 48.27, SD = 10.99, *N* = 32. The differences in terms of effect size were as follows: for context effects, *d* = 0.02, CI: from −0.52 to 0.56, v = 0.077. For music effects, *d* = 0.00, CI: from −0.57 to 0.58, *v* = 0.09. The results for the effects of musical background do not lend support to hypothesis 3 since the difference in accuracy between the two groups (music) and condition (context) failed to reach statistical significance, with *d* not approximating the threshold of 1.

With respect to RT, the differences between groups were as follows: *F*(2,101) = 128.040, *p* = 0.008 (NoContext Group musical background: M = 1367.46, SD = 546.52, *N* = 27; NoContext group no musical background: M = 1581.55, SD = 660.16, *N* = 25; Context Group musical background: M = 1399.79, SD = 655.24, *N* = 18; Context group no musical background: M = 1336.54, SD = 860.24, *N* = 32). The differences in terms of effect size were as follows: for context effects, *d* = −0.35, CI: from −0.96 to 0.246, v = 0.09. For musical background effects, *d* = 1.00, CI: from 0.4528 to 1.56, v = 0.08. Thus, context effects were weak in terms of effect size but quite robust for musical background.

It has to be noted that, unlike for accuracy, there was a difference between the two groups with regard to reaction time (*d* < 1.0). Thus, it appears that the subjects with musical backgrounds recognized reduced forms faster but not more accurately in comparison with subjects who received no such training. In light of the outcomes, hypothesis 3 assumes a more accurate and faster perception of reduced forms by learners with musical backgrounds in comparison with learners with no such background, which is negatively verified for accuracy and positively for reaction time.

## 4 Discussion

The study was geared to furnish answers to three research questions related to the acquisition of reduced forms by non-native speakers of English. Specifically, hypothesis 1 tested the effects of lexical context and was positively verified for native users only. Similarly, hypothesis 2, exploring the effects of a process type, proved correct for native speakers of English and showed weak or even very weak effects for learners of English. The effects of musical background, hypothesis 3, were not found in accuracy and were present in reaction time. These outcomes are discussed in turn.

We know that, in general, learners display a reliance on semantic context (e.g., Van De Ven et al., 2011) and that they require much more contextual information than native users (Nooteboom and Truin, 1980; Koster, 1987). In a perception study, Pearman (2004) also found that “learners required more acoustic information […] than natives in order to identify a word often needing to hear the beginning of the next word before recognizing an item correctly” (Pearman, 2004, p. 1). The present study, however, cannot provide support for the role of context for learners from analysis of reduced forms. The finding regarding the effects of semantic context is that it fails to improve the perception of reduced forms in SLA. This suggests that unlike other aspects of language acquisition, processes of casual speech pose considerable processing difficulties for learners, and the study argues that the inclusion of semantic context alone does not suffice to perceive reduced forms correctly by learners.

Although Polish learners of English were not sensitive to the effects of lexical context in recognizing reduced forms, they were influenced by the token duration and phone density. Phone density was calculated as the number of lexical items in the phrase or sentence that contained a reduced form. As a result, three groups emerged: low-density (between 3 and 5 words in the Context condition), mid-density (ranging from 15 to 16 words surrounding the reduced form), and high-density (25–39 lexical items preceding and following the reduced form in question). Low density was expected to facilitate correct identification, whereas high density, associated with increased cognitive load for learners, should prove difficult for perception. Figure 3 clearly points to a link between the number of words (token duration) and reaction time in both Context and NoContext groups of learners. In addition, within the Context group, phone density had a robust effect on learners whose perception deteriorated as phone density grew. Some effects of phone density are also visible in the NoContext group of learners, where too-short or too-long tokens were not correctly identified. Interestingly, mid-phone density had the strongest effect on perception without context. Following the results of hypothesis 1, the study thus argues that for Polish learners of English, the effects of phone density are stronger than the effects of lexical context.

Apart from undermining the role of lexical context for learners assumed in previous studies (its effects were observed for native users), the outcomes of the study throw certain doubts on the importance of typological differences. Its results are not in line with the findings of Shockey and Bond (2012) and Shockey and Ćavar (2013). They found that Polish learners recognized reduction processes very well and that they were well equipped to identify reduced forms in English, thanks to the high frequency of reduction processes in Polish. Figure 4 however, does not lend support to typological differences’ potential to explain learners’ performance. The lack of conformity between the results of the present study and the abovementioned ones might stem from methodological issues: Shockey and Bond (2012) and Shockey and Ćavar (2013) used read speech. This study employs a speech corpus of casual English. The two studies focused on those processes which perform consonantal cluster reduction, whereas the present study was more varied in the selection of processes (e.g., fricativization, Yod coalescence, and assimilation of place are non-reductory processes). Overall, the outcomes of the two processes that English and Polish have in their phonologies were mixed. Should typological differences be really significant, assimilation of place and t-deletion would be recognized in a more accurate fashion than the remaining four, more typologically distant processes. In light of the results (Table 4), this was not necessarily the case. For individual sounds, the Perceptual Assimilation Model (Best and Tyler, 2007) predicts that a category of a sound from L2 is assimilated to the nearest or equivalent category in L1. Categories ascribed to, say, a single vowel sound are usually two-dimensional within one phonemic unit (frontness and height).

Processes of casual speech are much more complex than individual sounds. A straightforward assimilation of a process in the first and second languages does not do justice to the complexity of reduction processes which involve, among others, a class of consonants affected by the processes, the impact of preceding and following sounds (place assimilation, Yod coalescence, and fricativization), position in a word (e.g., /t, d/ deletion), morphology (Yod coalescence), amount of stress and the grammatical status of a word (/h/ deletion), and many other variables. Thus, the study proposes that the transfer of phonological process categories present in L1 to L2 might be governed by entirely different mechanisms than those governing vowels or consonants. It is suggested that the transfer mechanisms may not fully account for processes of causal speech due to their structural dependence on larger units and spanning word boundaries. A similar conclusion was reached by Bradlow and Pisoni (1999): “In other words, spoken language processing relies on both accurate phoneme categorization and knowledge of the sound structure of the target language” (Bradlow and Pisoni, 1999, p. 2,084).

Another argument speaks in favor of the perceptual difficulty of reduced forms, which goes beyond the assimilation of a category. Vowel reduction, unlike consonantal reduction, is a well-established language universal (Greenberg, 1978) and is governed by grammar, stress assignment, and vowel quality, among other factors. Consonantally reduced forms, the subject of the study, have much less uniform patterns than vowel reduction. Some consonants become elided /t, d, h/, while others assimilate to their contextual neighbors (Yod coalescence and place assimilation) or to lenis consonants (fricativization). In addition, reduced forms occur both within and across word boundaries; sometimes, they become fully lexicalized (e.g., Yod coalescence in *gradual*). The latter, according to the Production Planning Hypothesis (Kilbourn-Ceron et al., 2020), have a wide array of constraints, such as conditional probability or phonological context, which may well add to the difficulties in recognition. This suggests that poor perception of reduced forms by Polish learners can possibly be accounted for by their high structural complexity which surfaces as perceptual difficulties.

There is also the issue of the frequency of occurrence of a process that is connected to linguistic typology. Maddieson (1984) found that in his database of 451 languages of the world, the CV structure is preferred: over 70% of world languages exhibit such preference and have no consonant clusters. In this connection, processes such as consonant cluster reduction are much more frequent in Polish than in English, and this exposure to high-frequency processes might at least in part explain the results obtained in the course of verification of hypothesis 1. The study makes a suggestion that the frequency of a process in L1 and L2 could be a good predictor for perception and perhaps would provide a reliable explanation of non-native perception. This suggestion remains speculative since research on casual speech is qualitatively oriented at the expense of the quantitative aspect, i.e., statistics on the frequency of realization of processes. For instance, Shockey (1974) states that “this process [place assimilation] is quite frequent in connected speech” (Shockey, 1974, p. 36). Lack of precision in these statements has already been pointed out by Watson (2006): “statements such as ‘plosives are frequently realized as affricates or fricatives’ are common in the literature, but it is never clear exactly how frequent frequent is” (Watson, 2006, p. 150).

The present study finds no effect of lexical context for learners, nor can it fully explain their perception using language-specific differences, especially if they are treated in binary terms of presence or absence of a process causing a reduced form. Alternatively, lack of exposure to casual English and difficulty with unfamiliar accents are proposed here as possible sources of influence. Typically, pronunciation courses in an L2 classroom rely heavily on sound articulation at the expense of listening activities. Drilling vowels and consonants takes up so much of the syllabus that very little time, if any at all, is devoted to perception. The use of a speech corpus in a classroom is also an infrequent practice. On top of that, there is little exposure to authentic casual speech: one may speculate that the instructors adapt their speech to language learners and extensively employ citation forms.

There is also a recurring issue with the pronunciation standard for SLA. Lancashire, a non-standard variety of English used in the study, is a northern English dialect. It exhibits the following features: monophthongization, t-to-r realization, /l/ vocalization and Yod dropping which label the dialect “non-standard” compared to the standard variety (i.e. SSBE) (Orton and Halliday, 1962; Wells, 1982; Beal, 2008). Therefore, students of English might experience problems in recognizing individual words on the one hand (though frequency counts prove that the words were not difficult, Appendix 1). On the other hand, for native speakers who represented various English accents (Table 2), Lancashire (with context) did not hinder understanding. We know that even very young children can recognize the meaning of words in non-native accents: Mulak et al. (2013) demonstrated that 19-month-old Australian children could identify words in Jamaican English, linking this to specific language skills rather than overall cognitive ability. In addition, some subjects of the study from the learners’ group indicated in the pre-study questionnaire that they had been working in the Northern UK and might have some familiarity with Lancashire or a neighboring dialect.

Turning to the effects of process type (hypothesis 2), the study reports very weak effects for the categorical type and weak effects for the gradient type within the learners group. On the other hand, the effects were significant for native speakers of English, which is in line with previous findings (Holst and Nolan, 1995; Hanique et al., 2013). This hypothesis was put forward because in articulation studies, processes that are complete (the categorical type) pattern in a different way than the incomplete, transient ones (the gradient type of phonological processes). Hypothesis 2 extends the famous stop–fricative metaphor to perception: “it is easier to run into a wall than to halt an inch in front of it” (source unknown, in Boersma, 1990) and assumes that categorical processes are more perception-friendly than the gradient ones. The study predicted that the loss of a segment (/t, d, h/ deletion) is perceptually more salient than the change of a sound into a new quality (place assimilation, Yod coalescence, and fricativization). According to Figure 5, the perception of gradient and categorical reduction proved to be equally difficult for learners, whereas no differences between process types were reflected in the perception of native speakers. In light of these results, it seems that the type of process (gradient vs. categorical) plays no role in the perception of reduced forms in SLA as far as Polish is concerned. This might suggest that SL listeners do not perceive the fine-grained phonetic detail of a process.

Following previous studies, the present study extended the influence of musical background on the perception of reduced forms regarding consonants (hypothesis 3) and found no such effects for second language learners. Although the effects of musical background were attested in reaction time, they did not exert any influence on the accuracy of non-native perception. Contrary to these findings, studies by Milovanov (2009), Salcedo (2010), Besson et al. (2011), Gordon et al. (2011), and Balčytytė-Kurtinienė (2018) found the effects of musical training and aptitude on the perception of rhythm, pitch, and vowel reduction. It has to be noted that the reduction of vowels has the potential to change the rhythm and the syllabic structure of vowels, to which people with musical talent have reacted as expected. The reduction caused by consonants, on the other hand, is not capable of changing rhythmic properties or general melody of an utterance (with a notable exception of h-deletion in an unstressed pronominal form, which is still strongly affected by associated vowel change and stress shift). The lack of effects of musical background on the non-native perception of reduced forms, reported in the study, can be linked to the generally low melodic impact of consonantal processes on speech.

Another noteworthy observation is that RTs were longer for native speakers in comparison with L2 learners in the context condition (Figure 2). The speed–accuracy trade-off (Standage et al., 2014; Huang et al., 2017) explains that responses are more accurate and slower if the experimental conditions focus on accuracy. If the conditions favor speed, the responses are less accurate and faster. The study’s instructions had no conditions highlighting either accuracy or speed. There was no time limit for typing the responses or pressing a Yes/No button. In addition, the instruction did not stress that participants should type exactly what they hear, nor should they type the full phrase. The lack of clear experimental focus could have possibly given rise to more variability in the obtained results, both for learners and the control group of native speakers.

The study addresses non-native perception of reduced forms using a wide variety of consonantal processes from a corpus of casual speech and fills the gaps identified in the Introduction section. The study tests the effects of lexical context, type of process, and musical background, drawing a number of conclusions and making a number of novel contributions to the field of SL perception. The first conclusion is that hypothesis 1 positively verified the effects of lexical context for the native perception of reduced forms but not for the learners. The study suggests that learners used cues from phone density instead of lexical context or typological differences between L1 and L2. There is also the possibility that language-specific differences might surface more vividly if the frequency of process occurrence is considered. Drawing on exposure to reduced forms, current classroom practice, and perhaps lack of familiarity with various dialects of English can be enumerated as additional factors shaping perception. The study speculates that the general complexity of phonological processes affecting consonants calls for exploring the role of awareness and metacompetence. The second conclusion is that the effects of a process type are insignificant for learners and robust for native users. It points to a greater role of phonetic details in the recognition of phonological processes than expected based on production studies. The final conclusion is that there are no effects of musical background on the perception of consonantal reduction for learners.

In general, the study contributes to linguistic theory by increasing our understanding of the effects governing non-native perception of casual speech. Of particular theoretical interest in this study is the overall implication that, apparently, the perception of reduced forms in a second language might proceed according to entirely different mechanisms than those governing the perception of vowels or consonants. In general, the effects tested (i.e., context, process type, and musical background) suggest that the role of awareness in the context of casual speech is greater than that of segments. Perhaps, reduced forms exist as independent representations in non-native phonology, not accessed as a deviation from citation form but as separate entities. The results of the study raise new research questions and prove that the perception of reduced forms is well worth pursuing further and deserves a more exhaustive treatment. It has to be noted that recognition of reduced forms is much more perceptually challenging than recognition of individual sounds or words as has already been demonstrated by the results obtained in this study for native and non-native speakers of English.

From a methodological viewpoint, the study demonstrates the utility of a speech corpus (PAC, Durand and Pukli, 2004) for investigating the recognition of reduced forms. The study, however, is not devoid of limitations. First, it tested the perception of only one variety of English, which happened to be a non-standard one. Second, the corpus used in the study had only nine speakers and totaled about 4.5 h of casual speech, which is a relatively small sample to mine reduction processes. The sample size could be enlarged and more varied in terms of speakers. The third shortcoming of the study might possibly stem from the study design itself: no data were accessible from the E-prime script, whether reaction time and accuracy come from the first or second hearing.

These limitations serve to outline several implications for further studies. One of them is to conduct a cross-linguistic investigation of reduced forms in a number of typologically related and unrelated languages (preferably with parallel reduction processes) to determine the role of typological differences or similarities. Another implication is to tease apart the effects of process type under carefully constructed experimental conditions. A possible research venue is to include accents other than Lancashire with a view of substantiating any claims about Lancashire’s difficulty or unfamiliarity and answering the question of to what extent processes of connected speech are dialect-specific. In addition, future studies might pursue the same research questions using an entirely different pool of subjects, such as learners of English for communicative purposes, immigrants, and professional musicians. Finally, the use of an eye-tracking research paradigm could take non-native perception to a new level.

## Data availability statement

The original contributions presented in the study are included in the article/Supplementary material, further inquiries can be directed to the corresponding author.

## Ethics statement

Ethical review and approval was not required for the study on human participants in accordance with the local legislation and institutional requirements. The participants provided their written informed consent to participate in this study.

## Author contributions

MK: Writing – original draft, Writing – review & editing.
