# Supplementary material for: Perception of reduced forms in English by non-native users of English
Source: Front Psychol. 2024 Apr 24;15:1305134. doi: 10.3389/fpsyg.2024.1305134 (PMC11076711; doi:10.3389/fpsyg.2024.1305134)
Supplement: Supplementary file 1 [file Table_1.DOCX]

Supplementary Material

# Supplementary Data

**Appendix 1: Stimuli without the context, along with frequency from the British National Corpus (number given for every word separately).**

these years (122527, 87531)

do you (267396, 661498)

last year (73564, 72143)

did you (134501, 661498)

first year (119153, 72143)

as you (646387, 661498)

words you (23354, 661498)

mind you (26837, 661498)

makes you (16184, 661498)

he knows yet (633413, 7939, 33500)

choices you (1712, 661498)

Cyprus you (677, 661498)

was younger (872620, 5201)

what do you (238621, 267396, 661498)

would you (242195, 661498)

husband you (10581, 661498)

what you (238621, 661498)

left me (44480, 127872)

don't get (267396, 447618, 94823)

went for six (45368, 867618, 29444)

because it's (99494, 1045013, 978459)

must be (69514, 643901)

couldn't (158325, 447618)

don't go (267396, 447618, 85983)

past five (25086, 39453)

it's a night (1045013, 978459, 2136923, 34119)

just (124905)

that's (1108428, 978459)

shifts (1152)

just get (124905, 94823)

can't really (229823, 447618, 45814)

didn't (134501, 447618)

it's not (1045013, 978459, 447618)

recently (11973)

find some of it 40455, 165421, 3009801, 1045013)

told people (34970, 119936)

small island (42738, 6417)

round (30459)

and Friday (2587880, 5341)

pounds (12032)

childminder (53)

sandwiches (854)

and things (2587880, 40472)

husband worked (10581, 12234)

secondary (4762)

husband Phil (10581, 1866)

friends (14358)

find myself (40455, 11874)

would have (242195, 455972)

but he (440934 633413)

got his (89430, 404811)

when he (207314, 633413)

to him (2565070, 152045)

get him (94823, 152045)

seen him (36014, 152045)

at home (516591, 49811)

until he (39571, 633413)

lent him (907, 152045)

we haven't (346949, 455972, 447618)

with him (650940, 152045)

I had (858211, 415007)

see her (112950, 301315)

would have (242195, 455972)

we have (346949, 455972)

so he (236884, 633413)

don't have (267396, 447618, 455972)

could have (158325, 455972)

seven (16878)

ankle (930)

that girl (1108428, 14383)

happen (8406)

wooden pole (3365, 1324)

good people (79878, 119936)

on my (717612, 145250)

unfair (1876)

done better (33998, 34289)

eleven plus (3633, 7586)

in my life (1914216, 145250, 54419)

good question (79878, 25487)

than me (143004, 127872)

would be (242195, 643901**)**

good grief (79878, 1353)

in Bolton (1914216, 571)

in fact (1914216, 36295)

should be (107822, 643901**)**

one minute (291900, 8520)

ten pound (19418, 6697)

like that (145999, 1108428)

obvious (8234)

working (28374)

absolutely (5619)

but (440934)

love it (21953, 1045013

obviously (10567)

pictures (5295)

like (145999)

worked (12234)

roadworks (143)

like my (145999, 145250)

fit in (7946, 1914216)

brought up (19410, 204973)

**Appendix 2: stimuli with the context**

but you've remembered it all these years

do you know

I've been there last year

did you get the people you wanted

did you get her

when you're in the first year

did you get a discount

as you got older

words you mean

mind you he's been thoroughly spoilt

it just makes you realize

I don't think he knows yet

what do you want

those are my career choices you know

if you go to Cyprus you can take

when he was younger

what do you mean

would you not like to live

I said to my husband you know

and he said what you're doing

somebody left me five hundred pounds

you don't get as many euros as you should

we went for about six weeks

from the front door because it's quite

but it must be a good job

I couldn't get back to the hospital

I don't go to France

half past five

well it's a night out

we have a cup of tea and just a chat

that's how I come to be here

they must be altered

working bit longer shifts

and you just get out of it

we can't really have like

whether he didn't hit the snooze switch

or an afternoon shift so

it's not a lot of people

my parents just sent me

we have actually been to France recently

but I find some if it very stressful

I told people not to buy me presents

office where my husband worked

small island i think it's only ten miles

and my dad is just round the corner

next Thursday and Friday

five hundred pounds cheaper than it ever will be

nearly two thousand pounds

after school it's a childminder

when he couldn't stand up

light tea set up light sandwiches

with those weekends and things like that

people my husband worked with

you could go to the secondary school

my husband Phil sometimes comes

couldn't live in that environment

a lot of friends

which I find myself saying a lot

with some friends we knew from school

at secondary school

I would have been as happy

but he has to hold it

he's got his arm made to plaster it

when he get his stuff

i can't speak to him

couldn't get him out of bed

last night I had a little bit of wine

I've never seen him since

she stayed at home full stop

i worked there until he closed down

and he's lent him

now we haven't got children and no dog

I was every afternoon with him

I had a lot

I still see her occasionally

i would have liked to have done but

we haven't seen them for a long time

so he said you'll have to take an exam

because you don't have an equity card

she could have been a French teacher

forty six to forty seven

then I've sprained my ankle

I think I could think it about that girl

I do mind if it's going to happen

that I had new wooden pole

working with some really good people

I couldn't turn him on my own that's why I always had to ask for help

it's unfair because you don't know

they should have done better

when I passed my eleven plus

and the acting I wanted more than anything at that point in my life

that's a good question actually

you know younger than me

the room would be so quiet

familiarity good grief that was a big word

If I came upon a lottery I would still live in Bolton

in fact he was

I should be a bit mad

one minute it's like all right and then the next minute

Mark has given Mike ten pound

places like that

she walked around without a bra and it was really obvious

I'm gonna be working basically

it was absolutely horrendous

but I mean

we love it

I was obviously

those are old picture

obviously i had a family to look after

everything absolutely everything I should do that tonight

so I was like all right

job where she worked

and I went working in the ropeworks

she was like my partner

a job that fit in with my family

my last daughter was brought up by my mother really
